# Phenome-wide functional dissection of pleiotropic effects highlights key molecular pathways for human complex traits

**DOI:** 10.1038/s41598-020-58040-4

**Published:** 2020-01-23

**Authors:** Anton E. Shikov, Rostislav K. Skitchenko, Alexander V. Predeus, Yury A. Barbitoff

**Affiliations:** 1 Bioinformatics Institute, Saint Petersburg, Russia; 2 City Hospital No. 40, Saint Petersburg, Russia; 3 All-Russian Research Institute for Agricultural Microbiology (ARRIAM), Saint Petersburg, Russia; 4 ITMO University, Saint Petersburg, Russia; 5 Department of Genetics and Biotechnology, Saint Petersburg State University, Saint Petersburg, Russia

**Keywords:** Genome-wide association studies, Genetics research

## Abstract

Over the recent decades, genome-wide association studies (GWAS) have dramatically changed the understanding of human genetics. A recent genetic data release by UK Biobank (UKB) has allowed many researchers worldwide to have comprehensive look into the genetic architecture of thousands of human phenotypes. In this study, we used GWAS summary statistics derived from the UKB cohort to investigate functional mechanisms of pleiotropic effects across the human phenome. We find that highly pleiotropic variants often correspond to broadly expressed genes with ubiquitous functions, such as matrisome components and cell growth regulators; and tend to colocalize with tissue-shared eQTLs. At the same time, signaling pathway components are more prevalent among highly pleiotropic genes compared to regulatory proteins such as transcription factors. Our results suggest that protein-level pleiotropy mediated by ubiquitously expressed genes is the most prevalent mechanism of pleiotropic genetic effects across the human phenome.

## Introduction

Over the recent decades, many advances have been made in uncovering the genetic architecture of human complex traits. Genome-wide association studies (GWAS), emerging at the onset of the millennium, have facilitated the discovery of susceptibility loci for both complex disease and quantitative traits (reviewed in Mills and Rahal^1^). Most recent GWAS for common phenotypes such as type 2 diabetes include up to 900,000 individuals and allow identification of numerous novel risk loci^2^. Many methods have been developed to interpret the GWAS signal on both single-variant and genome scale, including a group of specialized gene set enrichment tests^3–5^ aimed at discovery of biological pathways and mechanisms related to complex traits. Integration of GWAS and expression quantitative trait loci (eQTL) data has also shed light onto the role of gene expression in complex traits, and allowed for identification and validation of causal genes^6^.

Advances in biobanking, genetic data acquisition, and analysis have allowed first glimpse into the genetic architecture of multiple traits at once, with the UK Biobank (UKB) genetic dataset of 500,000 individuals being increasingly used for diverse studies in statistical genetics^7^. These included, for example, estimation of natural selection effects on complex trait-associated variation^8^ and development of trait-specific risk scores for identification of individuals at high risk for complex disease^9^. Genetic associations in the UK Biobank data have attracted the attention of researchers worldwide, and several efforts have been made to aggregate genetic association data across all traits^10–12^. These studies highlighted the complexity of the human phenome, and pinpointed notable loci with multiple associations (e.g., the MHC locus).

Genetic effects of the same locus on multiple traits, termed pleiotropic effects, are widespread in natural systems ranging from bacteria to humans and have attracted a lot of attention over the years^13^. Historically, researchers have mostly used model organisms to study pleiotropic effects of loss-of-function mutations and gene deletions that are generally expected to have larger effect on related traits^14,15^. These studies have produced many estimates of how widespread pleiotropy is, which ranged from 5% to 30%. While some argued that gene deletions are expected to generate variants with very high effects, and thus represent an estimate of the upper limit of observed pleiotropy, others have highlighted the fact that pleiotropic variants with strong effects might undergo even more stringent purifying selection than non-pleiotropic ones^16^. On the other hand, pleiotropy of individual common variants with weaker effects only became accessible to the researchers with the advent of the GWAS. This, in turn, would mean that we should be able to detect higher levels of pleiotropy among more common variants with lower effect sizes, including numerous noncoding and regulatory variants, which are the most prevalent in GWAS.

The role of pleiotropy in genetic architecture has been discussed for over 100 years^13^. Debates over how widespread pleiotropy is had started as early as 1930, and resulted in formulation of the hypothesis of universal pleiotropy^16^. The most stringent variant of this theory, strong hypothesis of universal pleiotropy (SHUP), implies that every variant influences every trait, which was quickly realized to be not supported by most experimental observations. Variations on the theme of weak hypothesis of universal pleiotropy (WHUP) predicted network-like structure of relationship between genetic architecture and complex phenotype space, which is now mostly accepted to be true. However, the extent to which pleiotropy influences complex traits remains a matter of debate. With the increasing number of studied traits the general definition of pleiotropy becomes useless. Indeed, as it was pointed out before, while the number of genes for an organism is finite, the number of traits can be increased indefinitely^16^. For this reason, it is important to separate the “true” biological pleiotropy from statistical and classification artifacts.

**Figure 1:**
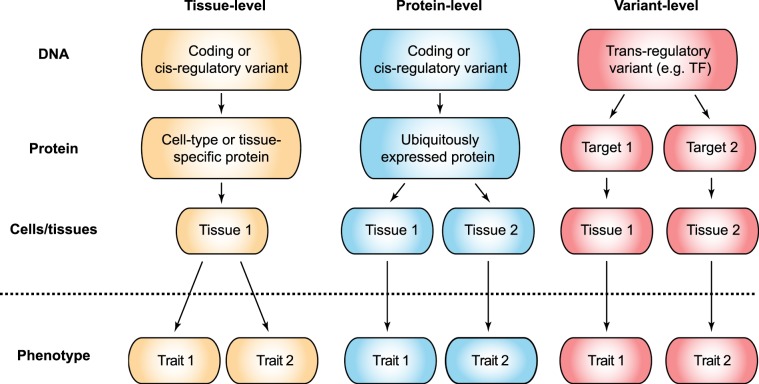
Three possible functional modes of pleiotropic genetic effects. Three situations that allow additional functional insights into pleiotropy: variant level, gene (protein) level, and tissue or cell type level pleiotropy.

There are several ways of classifying pleiotropic effects according to their mechanism. The first classification divides pleiotropic effects into three groups: (i) horizontal pleiotropy, with one variant independently affecting several phenotypes; (ii) vertical pleiotropy, when one of the traits is being mediated by the other; and (iii) spurious pleiotropy arising from one common marker variant being in close linkage disequilibrium (LD) with several independent causal variants^17^. Other authors suggest more mechanisms of cross-phenotype associations, involving regulatory effects of genetic variants, multiple gene deletions and pleiotropy mediated by different protein isoforms^18^. Given the availability of rich tissue-level expression datasets^6^, a useful way to classify pleiotropy in complex human traits might involve different levels at which pleiotropic effects might arise (Fig. 1). For example, multiple cross-phenotype associations might be driven by a single tissue with systemic impact on the whole organism (“tissue-level” pleiotropy); single protein with ubiquitous function (“protein-level” pleiotropy); and single variant affecting multiple genes (“variant-level pleiotropy”).

Pleiotropy of trait-associated variants in the human genome has attracted significant attention in the field^19,20^. Several attempts have been directed at studying pleiotropic effects of individual variants or genes across the whole phenome (phenome-wide association studies, PheWAS) (e.g., Schmidt *et al*.^21^), though no unified statistical framework to interpret such phenome-wide associations has been proposed. Mendelian randomization based approaches have also been proposed to detect pleiotropy in GWAS data^22^. The global picture of human pleiotropy, however, was obscured by differences in published studies, with many biases in phenotype and genotype data collection. UK Biobank dataset offers a unique opportunity to analyze shared components of the genetic architecture of uniformly processed human phenotypes on the genome-wide scale, with early global overviews suggesting widespread signals of pleiotropy in the UK Biobank data^23,24^.

In this study, we analyzed all heritable traits with reported associations using GWAS summary statistics from UK Biobank dataset, and assessed the prevalence and functional implications of different levels of pleiotropy.

## Results

### Complexity reduction by clustering of similar traits

To conduct a genome-wide scan for pleiotropic loci, we first obtained sets of significantly associated SNPs for all phenotypes in the UK Biobank data using pre-calculated GWAS summary statistics provided by Benjamin Neale’s lab (release 1, downloaded 2018-02-25). The dataset included both standard GWAS loci as well as imputed variants, totaling 10,894,597 variants. We focused our analysis only on 543 complex traits that have significant non-zero partitioned heritability estimates (h^2^). A total of 469,013 (4.27%) SNPs had at least one phenotype associated at genome-wide significant level, with an average of 4.34 phenotypes associated with each variant. Interestingly, we observed numerous multiple associations across the dataset, with 230296 (49.21%) of SNPs having >1 associated phenotype, and more than 10 associated phenotypes for 57,856 (12.34%) SNPs.

**Figure 2:**
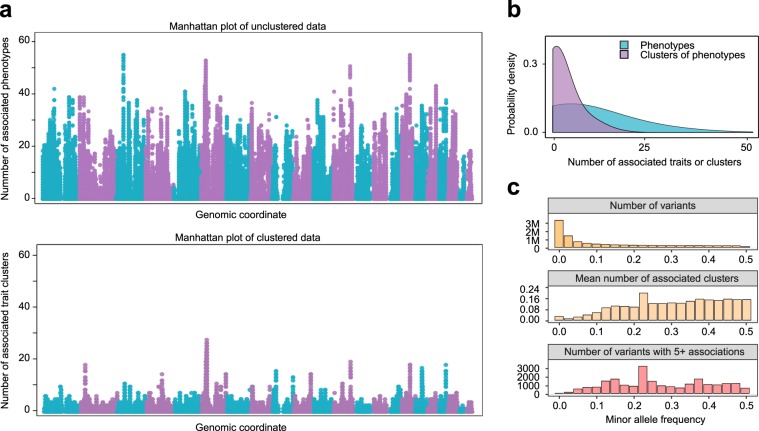
Clustering of similar traits significantly reduces the number of multiple associations. (**a**) Manhattan plot of the number of associations per SNP in the unclustered (top) and clustered (bottom) data. (**b**) Comparison of the numbers of associations per SNP before and after clustering of phenotypes by phenotypic correlation. (**c**) Summary statistics of associations for variants with different minor allele frequency. Values are aggregated over bins of size 0.025.

While many of these multiple associations could be true pleiotropic variants, much of the signal likely arises from multiple highly correlated phenotypes. Hence, we went on to cluster traits that share a significant proportion of their genetic architecture. We used reconstructed phenotypic correlation as the distance measure for clustering (see Methods). We applied hierarchical clustering combined with the analysis of silhouettes to determine the optimal number of independent clusters. We identified 308 clusters of complex traits including 1.76 phenotypes on average (annotated clusters available in Additional Data File 1 (see Methods)). Among these, 48 clusters comprised 3 or more phenotypes (see Fig. S1). The clustering procedure has substantially decreased the amount of SNPs having multiple associations (149,345 (31.1% of all associated) SNPs with more than one association compared to 230,296 in non-clustered data) (see Fig. S2); as well as the average number of associated trait clusters (1.77 clusters per variant). The number of pleiotropic SNPs with more than 10 associations has also dropped to 1.5% (7,072 variants) after performing clusterization procedure (Fig. 2a,b).

Interestingly, we observed a relatively small number of rare variants that have >5 associated clusters despite a much higher prevalence of rare variants in the dataset (Fig. 2c). In other words, rare variants tend to be less pleiotropic compared to common ones. Such differences in the degree of pleiotropy for rare and common variants can be explained in several ways (see Discussion).

### Phenome-wide analysis of gene set and pathway enrichment

We then focused on investigation of the genetic architecture of the major trait clusters. Many of the identified clusters comprised biologically similar complex traits or same phenotypes in different encoding (ICD-10 and self-reported disease status, etc.). In order to investigate the molecular basis of traits and clusters, we developed a simple modification of gene set enrichment analysis dubbed Locus Set Enrichment Analysis (LSEA) which accounts for linkage disequilibrium (LD) between SNPs and can work with few significantly associated SNPs for each trait (see Methods for description of the method). Our approach was more sensitive in identifying molecular pathways using a limited set of significantly associated variants, for example, for blood clotting-related traits (Figs. S3 and S4).

**Figure 3:**
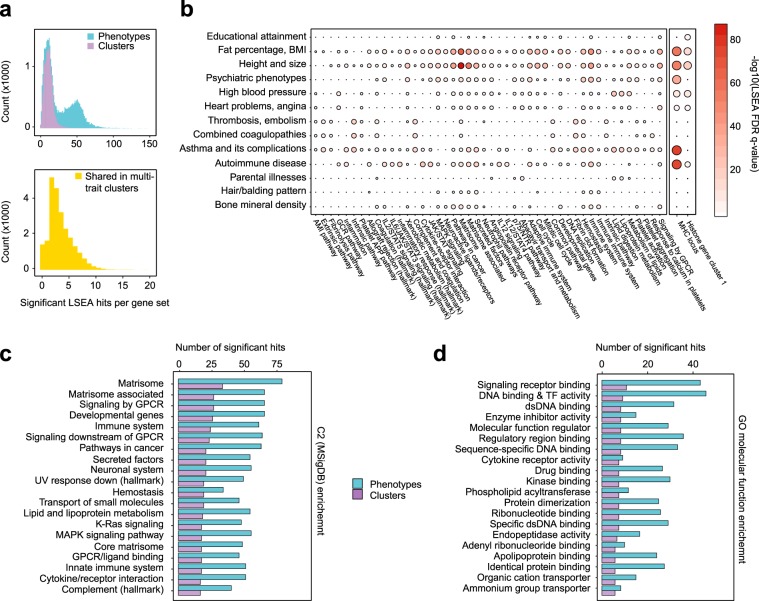
Complex trait clusters share several key enriched molecular pathways. (**a**) A histogram of numbers of enrichments per each gene set from the MSigDB collections. Upper panel, number of enriched phenotypes and enriched clusters; lower panel, number of clusters showing significant enrichment of 2 or more phenotypes. (**b**) A circle-map representation of LSEA results for common (shared by 2 or more phenotypes) associated SNPs for each multi-phenotype cluster. Only gene sets with shared enriched phenotypes in 2 or more clusters are shown (only top 100 enrichments for each cluster were chosen). (**c**,**d**) Curated gene sets (**c**) and GO molecular function terms (**d**) with the highest number of enriched clusters based on LSEA analysis. Top-20 hits are shown in each case.

We applied the LSEA method to make comprehensive functional annotations across the phenome using gene sets defined by Molecular Signatures Database (MSigDB)^25,26^. Overall, we observed substantial overlap between gene sets enriched in trait clusters and individual phenotypes (aggregated LSEA results available as Additional Data File 2). Out of 11,035 gene sets analyzed, 10,897 (98.7%) gene sets showed significant enrichment for at least 2 phenotypes from distinct clusters, with 2411 gene sets enriched in GWAS for more than 50 individual traits. (Fig. 3a). We also investigated whether a substantial proportion of gene set enrichments are shared in clusters that comprise multiple traits. Indeed, we found that 54% of gene sets enriched for at least one trait in multi-trait clusters were shared by at least one other trait in this cluster, with many of such shared gene sets also being associated to more than one clusters (Fig. 3a, bottom panel).

We then analyzed gene sets which were commonly associated with multiple trait clusters. To this end, we first selected top-100 enriched gene sets for each multi-trait cluster, and focused on gene sets having 2 or more enrichments after such filtering. Quite expectedly, we observed that most of the shared enriched gene sets belonged to the immune and metabolic pathways. However, we observed a very strong shared enrichment of extracellular matrix components that were enriched in 11 clusters (Fig. 3b). We also assessed the enrichment of association signal inside two large regions of chromosome 6, the MHC region and the histone gene cluster 1, which is known to harbor multiple binding sites for the master regulator of MHC genes, *CIITA*^27^. We found 8 trait clusters to be significantly associated with variation inside the MHC locus, supporting its pivotal role in complex traits. We next went on to assess the results of canonical pathway enrichment across the whole phenome (Additional Data File 3). On aggregate, matrisome and matrisome-associated proteins had the highest number of associated phenotypes and trait clusters (Fig. 3c). We also observed a high amount of associated phenotypes and clusters for genes involved in growth-related signaling (MAPK, K-Ras), immune and neuronal system function, and lipid metabolism. These results suggest that both tissue- and protein-level pleiotropic effects are prevalent, with commonly expressed genes such as matrisome component genes having a slightly higher degree of pleiotropy.

To investigate the role of variant-level trans-regulatory pleiotropic effects, we also analyzed the enrichment of Gene Ontology (GO) molecular function terms across all traits (Additional Data File 4). We identified that, while the strongest enrichment signal corresponded to signaling receptors, transcription factor genes were also commonly enriched in the association data. Interestingly, we observed that a slightly higher number of individual phenotypes showing enrichment for genes with DNA binding and transcription factor activity (Fig. 3d).

Overall, our analysis suggest that all three modes of pleiotropic effects described in Fig. 1 are present in phenome-wide associations. Having established that, we went on to dissect these functional effects by analyzing loci with different degrees of pleiotropy.

### Functional assessment of pleiotropic loci

**Figure 4:**
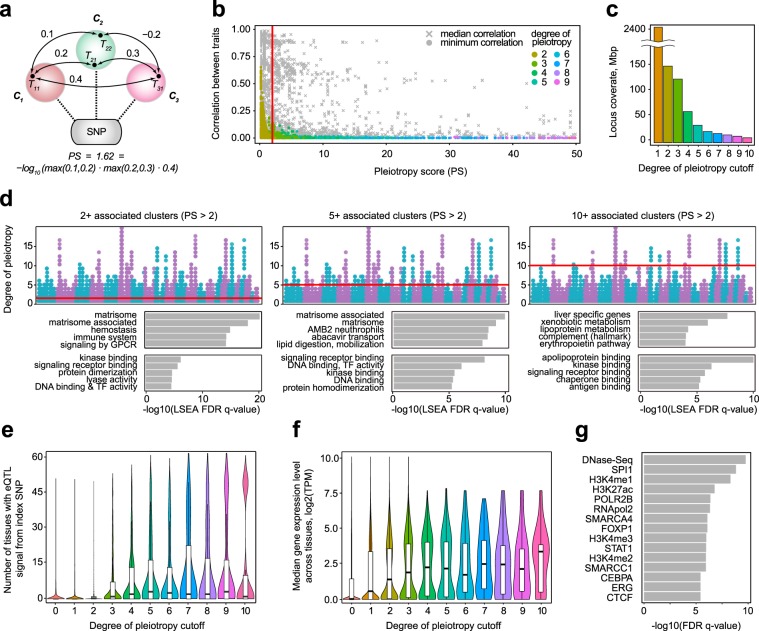
Pleiotropic loci comprise broadly expressed genes with ubiquitous functions. (**a**) A schematic for pleiotropy score correlation. In a typical example, pleiotropy score is calculated as log-transformed product of all pairwise cluster correlation coefficients. A correlation coefficient between two clusters is defined as a maximum correlation between traits that belong to each cluster. (**b**) A scatterplot showing the dependence of our pleiotropy score on median (gray crosses) or minimal (colored dots) cross correlation of associated clusters for each variant. Only variants with pleiotropy score of less than 50 and degree of pleiotropy of less than 10 are shown. Point color represents the degree of pleiotropy, the color code is identical to the one used in panels (с), (d), and (e). (**c**) Total length of loci with a given degree of pleiotropy cutoff as given by PLINK. (**d**) LSEA analysis of three groups of pleiotropic variants (filtered using total product correlation) - variants with 2+ associated clusters (left), 5+ associated clusters (middle), and 10+ associated clusters (right). Top-5 enriched genesets are shown. (**e**) Distributions of the number of tissues with significant eQTL signal for variant groups with different degree of pleiotropy cutoffs. (**f**) Distributions of median expression levels across GTEx tissues (log_2_(transcripts per million)) for genes located at loci with different degrees of pleiotropy. (**g**) Results of the ChIP-Atlas enrichment analysis^29^ of the pleiotropic loci vs blood-derived genomic tracks (see Methods). Top-15 enriched tracks are shown.

While clustering of phenotypes has alleviated most of the noise associated with multiple highly correlated traits, it is impossible to divide phenotypes into completely uncorrelated clusters. To this end, we developed a robust statistical score that allowed us to select “true” pleiotropic variants by accounting for the residual correlation between trait clusters. Briefly, we calculated a total product of pairwise correlation coefficients between clusters associated with the variant, and used this product as a proxy for the probability of the variant being deemed pleiotropic due to cluster correlation, *i*.*e*. vertically pleiotropic (see Methods). We then used log-transformed pleiotropy score (PS) to filter out loci with multiple associations to closely correlated clusters, such as height and body mass clusters (Fig. 4a). Removal of variants with PS ≤ 2 resulted in a set of 64,545 high-confidence “biologically” pleiotropic variants (results available as Additional Data File 5). For most of retained variants, there was at least one pair of traits that had correlation coefficient of 0.1 or less, while minimal correlation for most of the removed variants was substantially higher (Fig. 4b).

We next divided these pleiotropic variants into several categories by using different degree of pleiotropy cutoffs (2 and more clusters, 3 and more clusters, etc.) (see Supplementary Table S1 for a complete list of loci and variants). We first assessed the total length of the genome covered by pleiotropic loci with different number of associated clusters. Unlike previous reports^23^, we observed much lower coverage of the genome by pleiotropic loci, with 178.4 Mb covered by loci with 2 or more associated clusters and mere 11.6 Mb of loci with 10 or more associations (Fig. 4c).

We then applied LSEA to investigate molecular functions behind each category. Concordantly with the results obtained on a cluster-by-cluster basis, we identified the strongest enrichment of matrisome components and matrisome-associated genes in most categories (Fig. 4d, left and middle). The second most prevalent category of enriched gene sets were immunity-related pathways, which is also concordant with earlier observations (Fig. 3). Interestingly, we observed that matrisome genes are virtually absent among loci with the highest degree of pleiotropy (10 and more associated clusters), with this category including lipid and lipoprotein, and xenobiotic metabolism, as well as blood and immune cell signaling components. Analysis of GO molecular function terms revealed the strongest enrichment of signaling components, such as receptors and kinases, among each set of pleiotropic loci (Fig. 4d, bottom). Unlike in the cluster-by-cluster analysis (Fig. 3d), transcription factor genes showed much weaker enrichment compared to signal transduction genes.

Analysis of the functional impact of the variants with different degrees of pleiotropy have generated numerous intriguing results. We identified a very strong enrichment for cis-eQTLs among index SNPs in all variant categories (data collected from the Genotype Tissue Expression (GTEx); 586/2,106 for all index SNPs in pleiotropic loci compared to 5,640/59,449 for all index SNPs, Fisher p-value = 1.6 × 10^−131^), suggesting that variants with high pleiotropic effect tend to have a significant influence on gene expression. Interestingly, we observed that the degree of pleiotropy is positively correlated with the number of tissues with eQTL effect of the index variant (Fig. 4e) and the proportion of tissue-shared eQTLs (81/2,106 compared to 612/59,449 index SNPs matching eQTLs shared by more than half of studied tissues, Fisher p-value = 5.3 × 10^−21^). These results are in good concordance with other reports on genetic pleiotropy in the UKB dataset^23^, including a recent preprint by GTEx consortium^28^. Concordantly with these data, we found that genes at highly pleiotropic loci tend to be more broadly expressed across GTEx tissues (Fig. 4f), with the median expression level also increasing with the degree of pleiotropy. We also assessed the prevalence of different functional variant types in the set of pleiotropic variants. We found significant overrepresentation of missense variants (651/64,544 compared to 3,073/469,013, Fisher p-value = 3.1 × 10^−11^) among the pleiotropic variants compared to variants associated with at least one trait (Fig. S5). Furthermore, we saw strong enrichment for open and active chromatin marks (DNAse hypersensitivity regions, H3K4Me1, H3K4Me3, H3K27Ac, RNA Pol II) in the set of pleiotropic loci using genomic track enrichment analysis (ChIP-Atlas^29^, Fig. 4g). ChIP-Atlas analysis also identified enrichment of key transcription factor binding sites (*SPI*1, *STAT1*, *ERG*, *FOXP1*) within 1 kbp windows around index variants marking pleiotropic loci.

## Discussion

Several attempts have been previously made to assess pleiotropic effects in human complex traits based on GWAS data^19,20^. Such studies used GWAS Catalog data^30^ or electronic health records^31^. These studies highlighted widespread pleiotropy and pinpointed most notable pleiotropic variants, but could not offer functional and statistical characterization of pleiotropy in human genome due to limits of the datasets. Using UK Biobank data, Watanabe *et al*. have recently demonstrated some of the key features of pleiotropic variants^23^. However, the use of sparsely defined trait domains instead of statistically defined trait clusters led to very high estimates of pleiotropy, with 142,439 out of 236,638 (60%) significantly associated SNPs reported as pleiotropic. The loci that contained pleiotropic SNPs were estimated to cover as much as 1.6 Gb of human genome.

In a different approach, Jordan *et al*. have recently developed HOPS, a statistical “whitening” procedure that removed trait-based correlation and allowed robust identification of “biological” pleiotropy^24^. Additional modelling of polygenicity of each trait makes the proposed model especially attractive for functional dissection of pleiotropy. Authors report a much smaller number of pleiotropic SNPs, with the biggest estimate being 74,335 variants in 8,093 LD-independent loci. While this makes pleiotropy still very widespread, it does not quite reach the levels described in Watanabe *et al*. We have independently developed another statistical score that allowed us to filter the variants associated with highly correlated clusters, e.g. large clusters that included most of the anthropometrical traits. Our estimates (64,545 variants in 1,315 loci) fall much closer to *Jordan et al*., especially taking into consideration that our method of pleiotropic SNP identification can only discover SNPs that are significantly associated with at least two traits in a univariate GWAS, while HOPS allows for discovery of novel associations^24^. We estimate approximately 180 Mbs of the human genome to be covered by pleiotropic loci; the exact coordinates and included SNPs can be found in Supplementary Table S1.

We show that pleiotropic effects are much more widespread in common variants than in rare variants (Fig. 2c). This observation might be explained in three possible ways. Firstly, lack of rare pleiotropic variants might be caused by limited statistical power to detect association for rare variants. Secondly, higher prevalence of common pleiotropic variants might suggest that much of their pleiotropic effects are in fact spurious and are explained by high LD to several distinct causal variants^17^. Finally, lower degree of pleiotropy of rare variants might be caused by strong purifying natural selection acting against highly pleiotropic variants with larger effects, causing all pleiotropic variants to have lower effect sizes and higher frequency. Negative natural selection was shown to operate on trait-associated variation^8^, favoring the latter mechanism.

To investigate the exact molecular functions of highly pleiotropic genes, we made a comprehensive functional annotation of GWAS signal for individual traits and trait clusters across the phenome. To perform such functional annotation using a limited set of significant variants, we developed an LD-aware variant of GSEA we called LSEA, which allowed us to identify crucial molecular pathways influencing multiple clusters of phenotypes. We discovered that genes with the highest degree of pleiotropy are involved in matrisome organization, cell cycle, and immunity (Fig. 3b–d). Broad influence of immune system on complex traits is widely acknowledged, and similar observations about high degree of pleiotropy for genes involved in immunity (including HLA genes) have been made previously using site-level PheWAS^31^. At the same time, an overwhelming prevalence of matrisome component genes among pleiotropic loci (Figs. 3c,d and 4d) has not been reported previously.

Matrisome comprises a set of extracellular molecules that form and regulate the extracellular matrix (ECM) that governs the structure of most tissues. ECM proteins have been shown to have a significant role in body composition and in various diseases^32^. These included both complex diseases as well as different types of cancer^33^. Matrisome itself consists of both core ECM proteins (core matrisome) and matrisome-associated proteins and regulators (mostly composed of signaling molecules and secreted metabolic factors). Notably, we find that the core matrisome components are less pleiotropic compared to matrisome regulators/matrisome associated genes (Fig. 3c). This suggests that variation in the core elements of the ECM might have a stronger effect on the phenotype, manifesting in monogenic disease rather than complex phenotypes; at the same time, subtle variation in ECM structure due to regulatory alterations is important for complex traits.

Such unusually high prevalence of matrisome components and cell growth regulators in GWAS signal across the phenome highlights the strongest influence of protein-level pleiotropy on human complex traits (Fig. 1). This assumption is further corroborated by a strong enrichment of tissue-shared eQTLs (Fig. 4e) among index SNPs with higher degree of pleiotropy, as well as higher median expression level of genes located at highly pleiotropic loci (Fig. 4f). Similar observations have been made using slightly different approaches in other overviews of pleiotropic effects^23^; moreover, a recent analysis of regulatory variation by the GTEx Consortium suggested that trait pleiotropy might also be connected to the “cis-eQTL pleiotropy”, *i*.*e*. regulatory effects of a single variant on multiple neighboring genes^28^.

Importantly, our data on pleiotropic effects of certain key loci corroborate and expand previous results obtained using Mendelian randomization approach^34^. For example, we find additional phenome-wide associations for previously reported pleiotropic genes, such as *FTO*, *APOE*, and *SLC*3*9A8*. We also discovered several notable examples of novel pleiotropic loci. One such example is the *MIR2*1*13* gene encoding a microRNA of unknown function. This locus is associated with several phenotypes related to cognitive characteristics, including college or university degree qualifications, fluid intelligence score, as well as urine ion concentrations (sodium and potassium). Previously, *MIR*2*113* was shown to play a role in memory processes^35^. However, the exact mechanism of its function has never been reported. Our data on its involvement in maintenance of ionic balance, together with a strong enrichment for potassium channel genes among its predicted targets according to miRBase (Fig. S6), suggest that *MIR*2*11*3 affects cognitive processes through regulation of ion channel gene expression.

Overall, our approach allowed us to detect multiple novel interrelations between complex traits, which can shed light on common molecular patterns driving the human phenome, as well as underlying mechanisms of diseases with unknown etiology^20^. Further investigations would help determine causal relationships between key genes and molecular pathways that give rise to polygenic disease. Investigation of all possible genetic interactions between common and specific risk factors should help to decipher sophisticated molecular mechanisms behind complex traits.

## Methods

### Data acquisition and filtering criteria

We acquired data from the Benjamin Neale’s lab website (UK Biobank GWAS results imputed v2, downloaded at 2017-10-04; http://www.nealelab.is/uk-biobank). We focused our attention only on phenotypes with significant non-zero partitioned heritability estimates (p < 0.05, estimates provided by the authors of the dataset). After obtaining the list of heritable phenotypes we retained genome-wide significant variants for each phenotype (p-value < 5 × 10^−9^). Association summary statistics for each variant for each trait were then merged into a single matrix for further analysis.

### Clustering of traits

To group similar traits into clusters, we utilized the reconstructed phenotypic correlation which was calculated from summary statistics using the PhenoSpD toolkit^36^. We then performed hierarchical clustering using the absolute value of such phenotypic correlation. Optimal number of clusters was selected using the analysis of silhouettes. To calculate the number of associated trait clusters for each variant, we considered a SNP to be associated with a cluster if this SNP is associated with at least one trait belonging to this cluster.

### Description of the LSEA method

We implemented a novel Locus Set Enrichment Analysis (LSEA) method for gene set enrichment analysis of GWAS data. LSEA operates on the sets of pre-selected significant SNPs or summary statistics file for each trait. At first stage, LSEA groups associated SNPs into individual genomic loci. First, variants are clustered by LD using the clumping method in PLINK^37^. The resulting groups of SNPs are transformed to an interval list based on the leftmost and rightmost coordinate of variants in each clump. The resulting intervals are then merged if these overlap by more than 70%, or span different parts of the same gene, and intervals overlapping the MHC region or the *HIST*1 gene cluster (defined as chr6:28,866,528-33,775,446 and chr6:25,000,528–28,000,446, respectively, based on hg19 human genome annotation) are omitted, as suggested previously^3^. The resulting intervals are then intersected with the pre-computed universe of all possible loci bearing GWAS signal.

To construct such universe, we merged all loci that were identified for the whole UKB dataset. We then transformed all of the curated gene sets obtained from the MsigDB database (http://software.broadinstitute.org/gsea/msigdb/annotate.jsp) the following way: for each interval *i* in the intervals universe, we assigned *i* to a gene set if at least one gene in the gene set overlaps *i*. Such method efficiently corrects for functionally related genes that are genetically linked, as intervals spanning several genes belonging to each gene set are counted only once.

The enrichment statistic is computed for each gene set as follows:

$$F=\mathop{\sum }\limits_{j=1}^{n}\,{i}_{j}$$

where *n* is the number of intervals in the query set, *i*_*j*_ = 1 if interval *j* belongs to a gene set of interest, and *i*_*j*_ = 0 otherwise. Enrichment p-value is then computed from hypergeometric distribution. The resulting p-values are then adjusted for multiple comparisons using the Holm FDR correction.

### Gene set enrichment analysis of individual traits and clusters

We used the LSEA method to analyze gene set enrichment for all individual phenotypes and trait clusters. For the analysis of multi-trait clusters, we used LSEA results for all individual phenotypes in each cluster, and retained only gene sets that were significantly enriched for 2 or more traits in a cluster (termed “union of gene sets”).

As the HLA locus and the *HIST1* gene cluster were omitted during gene set analysis with LSEA, we analyzed the overrepresentation of variants inside these loci separately. To this end, we created sets of intervals for the *HIST1* and HLA regions in a way analogous to the LSEA method. We then obtained the overrepresentation p-values using hypergeometric distribution, with the test statistic being equal to the number of intervals in the query that overlap the regions of interest.

### Identification of “biologically” pleiotropic genetic variants

In order to reduce the influence of trait correlation, we have clustered 543 traits with non-zero heritability into 308 clusters. However, some distinct pairs of clusters still contained substantially correlated traits, leading to incomplete separation of vertical pleiotropy. To address this, we developed a pleiotropy score that characterized the probability of a particular SNP being associated with two or more clusters of traits simply due to traits being correlated.

Relationship between correlation coefficients lacks transitivity; in fact, for correlation between three vectors, it can be shown that there are limitations which values c(*v*_*1*_,*v*_*3*_) can take only when c(*v*_*1*_, *v*_*2*_)^2^ + c(*v*_*2*_, *v*_*3*_)^2^ ≥ 1^38^. The issue becomes more complicated with for 4 and more vectors involved. Using correlation between clusters of traits, we can eliminate the necessity to account for independence of traits. We defined the upper limit of correlation between two clusters as the highest absolute value of correlation between any pair of traits *T*_*i*_, *T*_*j*_, where *T*_*i*_ belongs to cluster *C*_*n*_ and *Tj* belongs to cluster *C*_*m*_. After this, the probability a SNP being pleiotropic due to simple correlation of phenotypes included in clusters is calculated by multiplication of correlation of all possible pairs of clusters. That is, if a SNP is associated with N clusters (*C*_*1*_*…C*_*N*_), probability score is calculated as

$$PS=-\,{\log }_{10}\mathop{\prod }\limits_{n=1,m=1,n < m}^{n=N,m=N}\,corr({C}_{n},{C}_{m})$$

where correlation between clusters is defined as

$$corr({C}_{n},{C}_{m})=\mathop{\max }\limits_{{T}_{i}\in {C}_{n},{T}_{j}\in {C}_{m}}|corr({T}_{i},{T}_{j})|$$

Using pleiotropy score we can filter off all variants which have a high chance of being vertically pleiotropic. Figure 4b shows the behavior of minimal and median correlations between all the traits included in associated clusters, indicating that PS of 2 is enough to filter off most vertically pleiotropic variants, while still keeping variants associated with 2 orthogonal clusters.

### Overrepresentation analysis for functional variant classes

To analyze the overrepresentation of cis-eQTLs, we used the GTEx consortium dataset^28^. Sets of variants of interest (i.e., pleiotropic variants and variants with at least 1 association) were overlapped with significant cis-eQTLs (p < 5 × 10^−8^). Overrepresentation of cis-eQTLs in the set of pleiotropic SNPs was assessed by comparison of the resulting proportion of significant eQTLs using the hypergeometric distribution.

We also annotated all SNPs from the UKB dataset with SnpEff^39^ and separated the variants by their functional type (e.g., missense, intronic, etc.; the distributions are shown in Fig. S5). We then compared the proportions of certain functional classes of variants in the sets of pleiotropic SNPs and SNPs bearing at least one association. The proportions were compared using the hypergeometric test.

To analyze the enrichment of epigenetic marks and chromatin states in the set of pleiotropic loci, we used the previously described ChIP-Atlas server^29^. For this analysis, sets of pleiotropic variants were converted to an interval list, and the resulting intervals were used as the input for ChIP-Atlas. To narrow down the search space, we limited the analysis down to a set of blood-derived genomic tracks. For each type of epigenetic mark, we retained the minimal enrichment p-value for further analysis.

## Supplementary information

Supplementary Figures S1-S6.

Supplementary Table S1.

## Data Availability

All scripts pertinent to the analysis presented here, as well as the source code for LSEA, are available through GitHub (https://github.com/bioinf/ukb_phewas). Additional Data Files referenced in the manuscript can also be obtained from the GitHub repository.
