# Supplementary material for: Phenome-wide functional dissection of pleiotropic effects highlights key molecular pathways for human complex traits
Source: Sci Rep. 2020 Jan 23;10:1037. doi: 10.1038/s41598-020-58040-4 (PMC6978431; doi:10.1038/s41598-020-58040-4)
Supplement: Supplementary file 1 — Supplementary Figures S1-S6. [file 41598_2020_58040_MOESM1_ESM.pdf]

Phenome-wide functional dissection of pleiotropic effects highlights key molecular pathways for human complex traits

Anton E. Shikov[1,2,3], Rostislav K. Skitchenko[1], Alexander V. Predeus[1*], Yury A. Barbitoff[1,4*]

[1] Bioinformatics Institute, Saint Petersburg, Russia

[2] City Hospital No. 40, St. Petersburg, Russia

[3] All-Russian Research Institute for Agricultural Microbiology (ARRIAM), Saint Petersburg, Russia

[4] Dpt. of Genetics and Biotechnology, Saint-Petersburg State University, Saint Petersburg, Russia

[*] These authors jointly supervised the work. Correspondence: barbitoff@bioinf.me, predeus@bioinf.me

Keywords: phenome-wide association study, pleiotropy, molecular pathway, complex trait, eQTL, matrisome pathway, complex trait

Supplementary Information

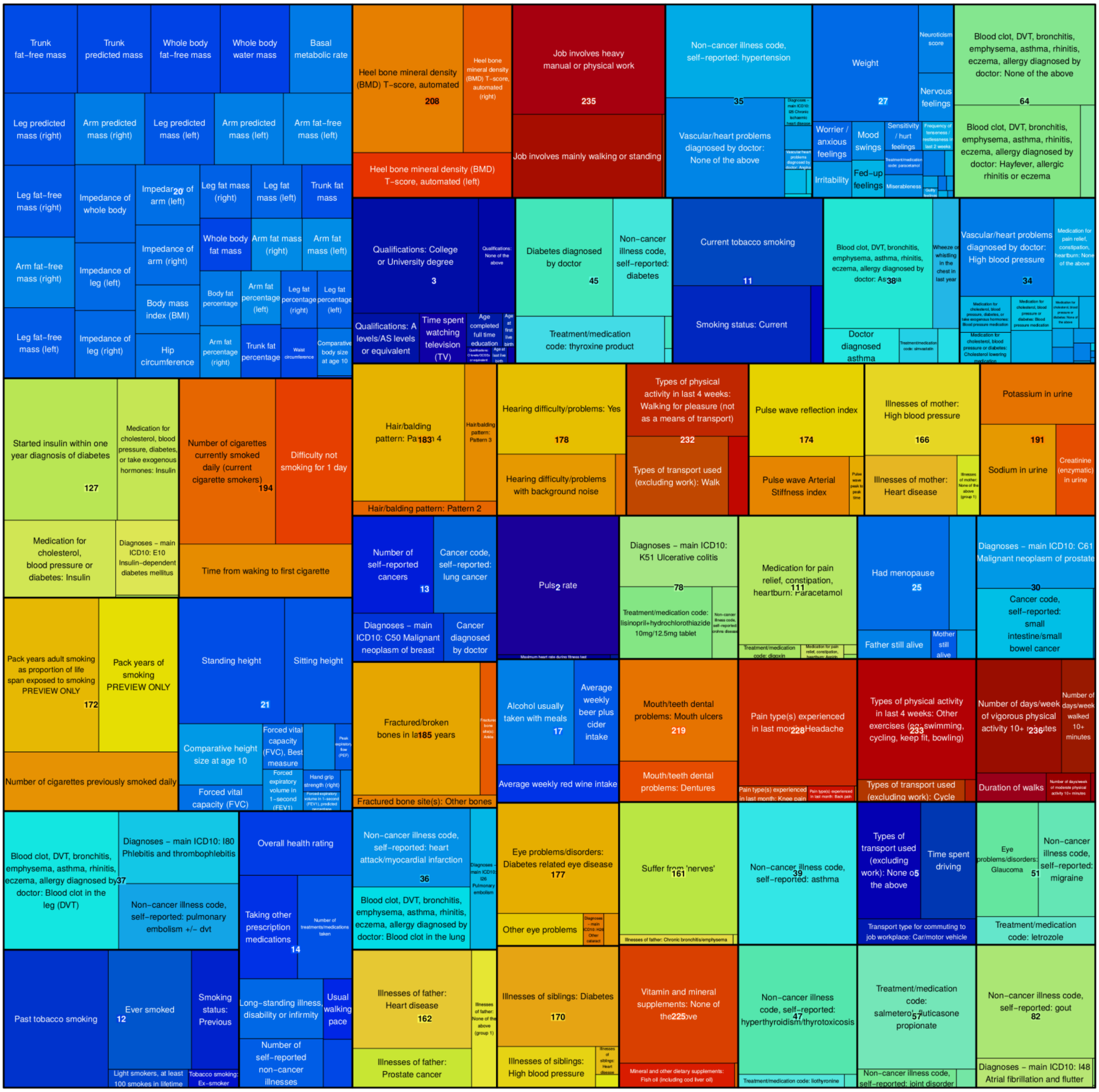

Figure S1. Voronoi treemaps of multi-phenotype clusters defined by hierarchical clustering of the significant SNPs. Color blocks correspond to individual clusters. Numbers are equal to the number of cluster used throughout the work.

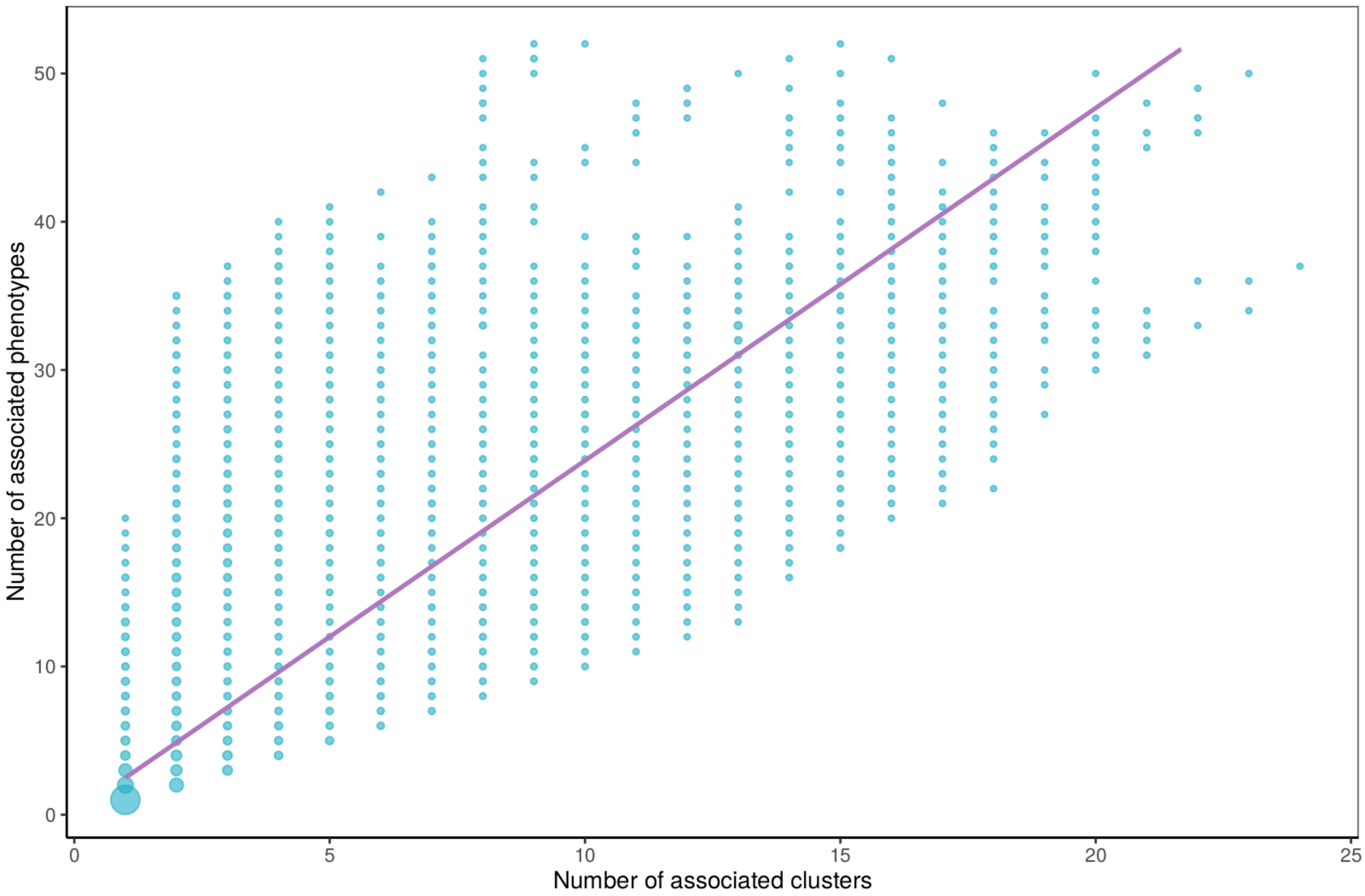

Figure S2. A scatterplot of the per-SNP number of associated phenotypes and phenotype clusters. Dot size is proportional to the log10 (number of SNPs).

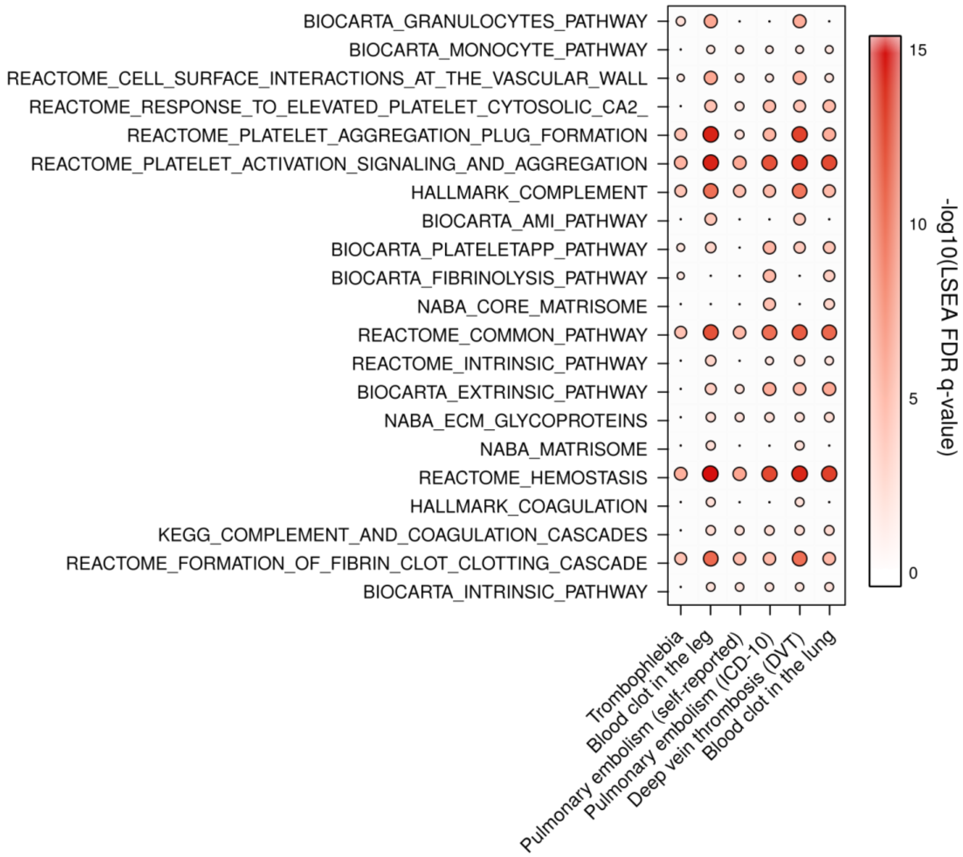

Figure S3. LSEA successfully identifies relevant molecular pathways for blood clotting-related phenotypes (clusters 37, 38).

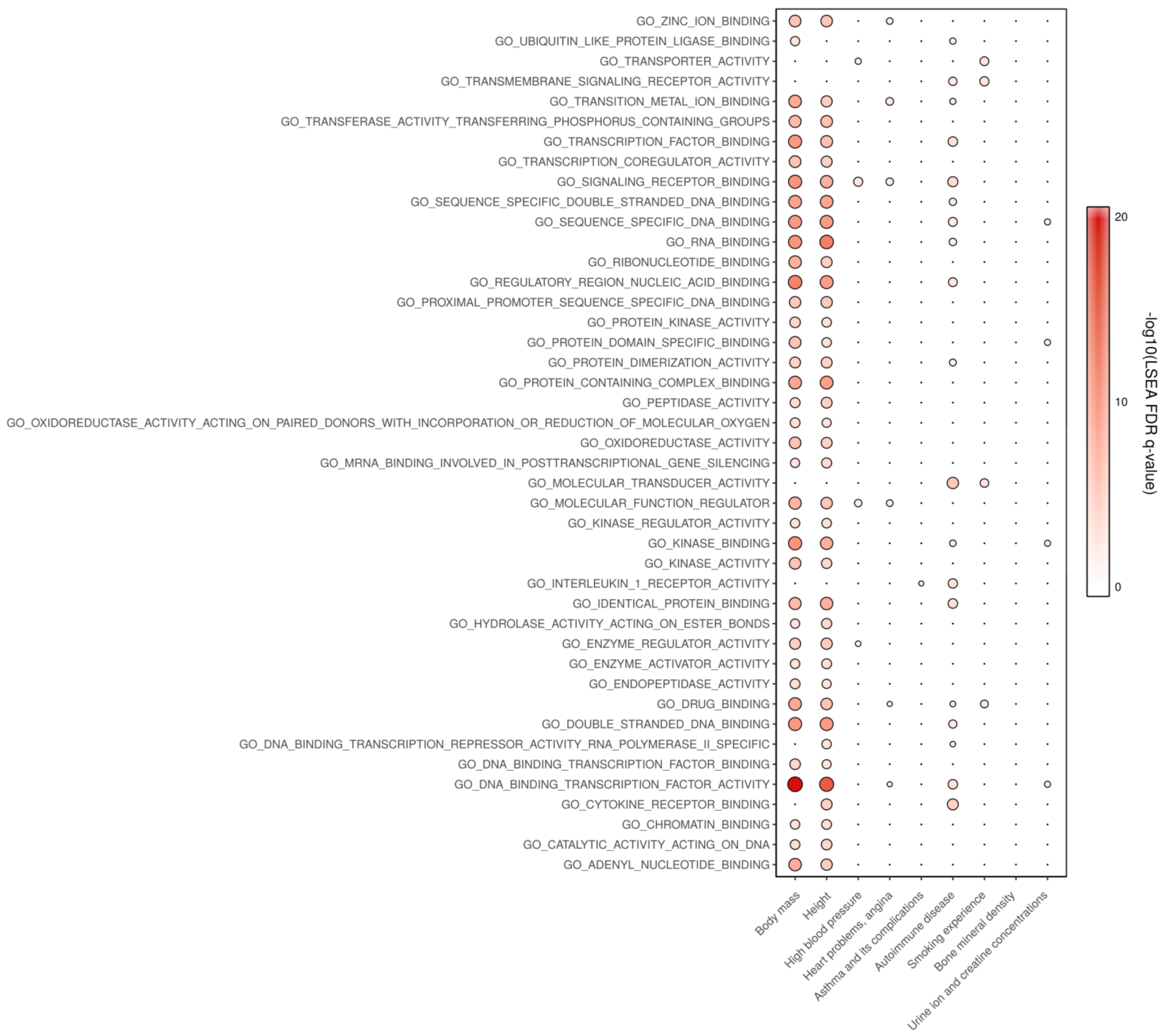

Figure S4. LSEA analysis identifies key GO molecular functions enriched for major trait clusters. Only trait clusters showing at least one enriched GO molecular function term are shown. Note that transcription factors shows the highest prevalence.

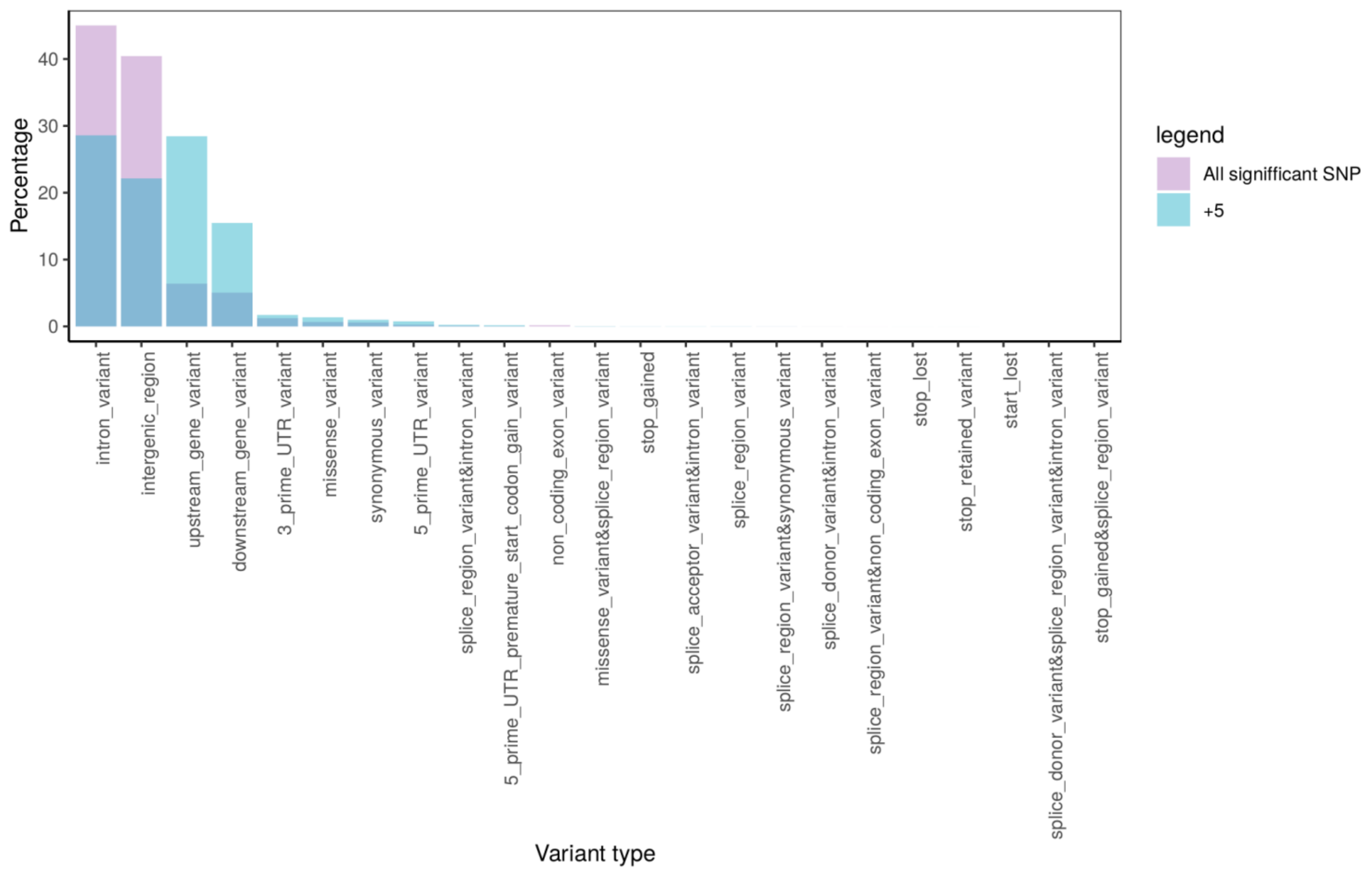

Figure S5. A bar plot of the fraction of SNPs in each functional class for pleiotropic SNPs and all SNPs with at least 1 significant association. Upstream, downstream, and missense variants are overrepresented in the pleiotropic group (results are concordant with the ones reported in Watanabe et al., 2018)

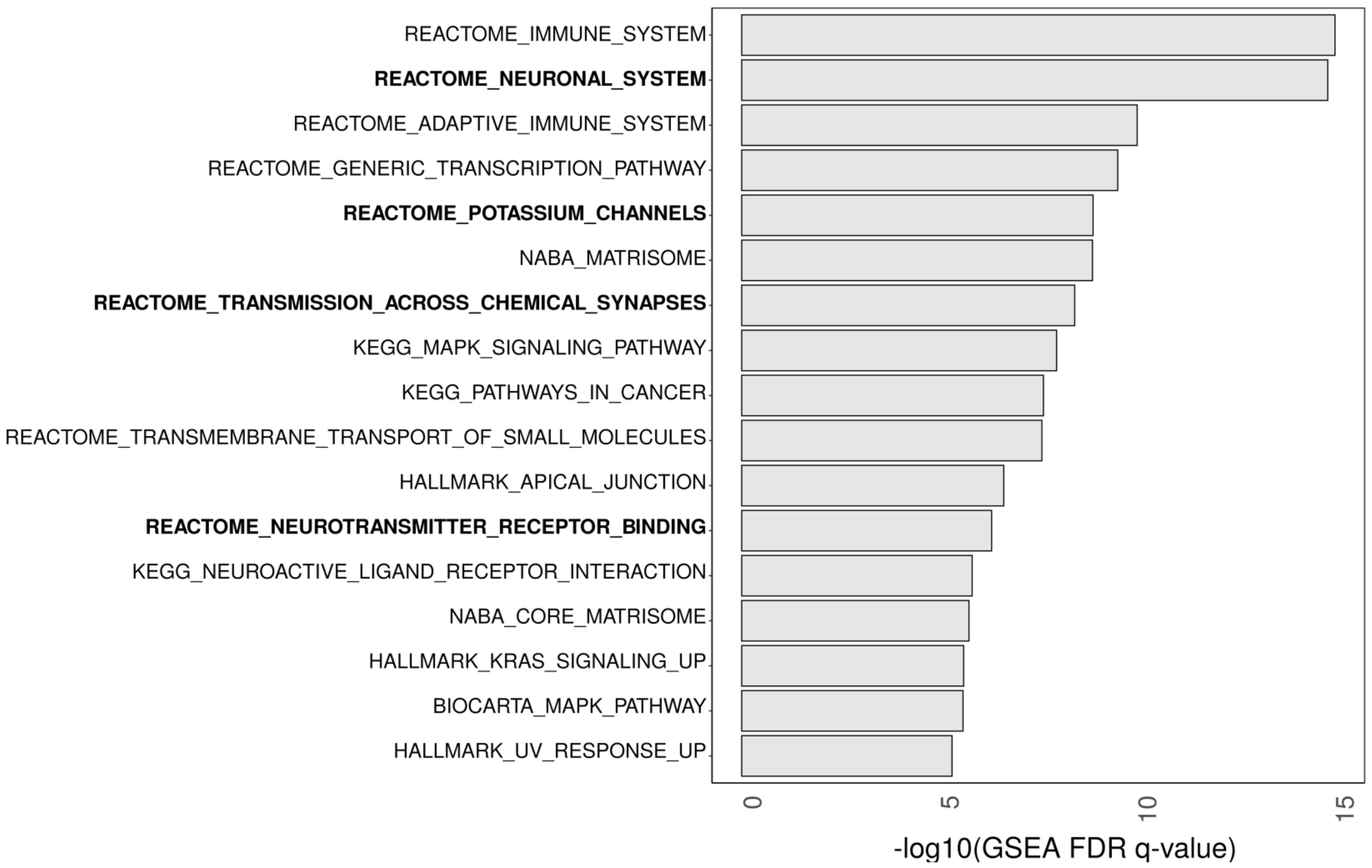

Figure S6. Results of the Molecular Signatures Database (MSigDB) GSEA analysis of top-2,000 targets for the *MIR2113* microRNA (as reported in the miRBase TargetScan results). Top 10 enriched gene sets are shown. Gene sets relevant to nervous system function are highlighted in bold.
